# The Effect of Antiretroviral Combination Treatment on Epstein-Barr Virus (EBV) Genome Load in HIV-Infected Patients

**DOI:** 10.3390/v2040867

**Published:** 2010-03-29

**Authors:** Anna M. C. Friis, Katarina Gyllensten, Anna Aleman, Ingemar Ernberg, Börje Åkerlund

**Affiliations:** 1 Microbiology and Tumor Biology Center, Karolinska Institute, SE-171 65 Stockholm, Sweden; E-Mails: anna.friis@ki.se (A.M.C.F.); anna.aleman@ki.se (A.A.); 2 Gay Men’s Health Clinic, Karolinska Institutet, Department of Clinical Science and Education, Södersjukhuset, SE-118 83 Stockholm, Sweden; E-Mail: katarina.gyllensten@sodersjukhuset.se (K.G.); 3 Department of Infectious Diseases, Karolinska University Hospital, Huddinge, SE-141 86 Stockholm, Sweden; E-Mail: borje.akerlund@karolinska.se (B.Å.)

**Keywords:** Epstein-Barr virus, HIV-1, antiretroviral treatment, host virus relationship

## Abstract

We evaluated the effect of combination anti-retroviral treatment (cART) on the host control of EBV infection in moderately immunosuppressed HIV-1 patients. Twenty HIV-1 infected individuals were followed for five years with repeated measurements of EBV DNA load in peripheral blood lymphocytes in relation to HIV-RNA titers and CD4+ cell counts. Individuals with optimal response, *i.e.* durable non-detectable HIV-RNA, showed a decline of EBV load to the level of healthy controls. Individuals with non-optimal HIV-1 control did not restore their EBV control. Long-lasting suppression of HIV-replication after early initiation of cART is a prerequisite for re-establishing the immune control of EBV.

## 1. Introduction

Before the introduction of effective combination anti-retroviral treatment (cART) of HIV-1 infection in 1996, HIV-infected patients had an over a 1000-fold increased risk of developing non-Hodgkin lymphomas (NHL) [1,2]. Such an increased risk of NHL was previously observed among post-transplant patients [3,4]. The pathogenic role of Epstein Barr virus (EBV) in the development of those malignancies is of great concern. In HIV-1 infected individuals, almost all primary central nervous system lymphomas are EBV positive [5,6]. In contrast, HIV-associated peripheral lymphomas display a lower frequency of EBV positivity [7]. EBV latency associated genes, Epstein-Barr nuclear antigens (EBNA)-1, EBNA-2 and latent membrane protein (LMP) have been expressed in varying frequencies in the lymphomas of HIV-infected patients. The role of disrupted immunosurveillance and T-cell immunity in the control of EBV replication was shown early in the epidemic [8]. After the introduction of cART, a general improvement of immune function in HIV-infected patients was observed, together with a significant decrease of NHL [9]. In AIDS patients, an association between EBV DNA load and the loss of EBV-specific CD8+ T cell function has been demonstrated [10]. There is also a correlation between EBV DNA load, numbers of CD4+ cells, and numbers of functional T-cells [10].

During the pre-cART period, we analyzed EBV load in several subgroups of HIV-infected patients. A particularly prominent increase in EBV load (40–50-fold) was seen in patients participating in a placebo controlled trial with recombinant gp-160 vaccine. Twenty HIV-patients with moderate immune deficiency, who had previously taken part in this trial [11], were regularly tested during a five year follow up. The virological and immunological effects of cART measured by HIV-1 RNA titers and CD4+ cell counts were analyzed in relation to EBV load.

## 2. Results and Discussion

In the samples from HIV-infected patients, the EBV load varied considerably with a range between 0.003 to 6700 EBV DNA copies per 1000 B-cells (approximately 250,000-fold difference). The intra-individual variation was much lower (approximately 2500-fold difference). Four patients showed a very high EBV load (>100) on at least one occasion. In contrast, all repeated values were below 0.6 EBV copies per 1000 B-cells (median 0.05: range: 0.01–0.6) in 10 HIV-negative controls. The intra-individual variation seen in the control group was less than two-fold.

Based on the HIV-1 RNA levels, there were three main patterns crystallized regarding the patients virological and immunological response to cART and the impact of the treatment on the EBV load. Group I showed HIV-1 RNA levels constantly below the detection limit after initiation of cART, indicating optimal inhibition of HIV-1 replication. Patients of group II had most but not all HIV-1 RNA levels below the detection limit; while in group III almost all values were above the detection limit. There was no difference in the frequency of HIV-RNA measurements between the three groups. Three patients were lost to extended follow up and one patient was treated only with a single NRTI-drug. These patients were excluded from this analysis. There were no differences in gender, median age, route of transmission or ethnicity between the groups.

**Figure 1.**
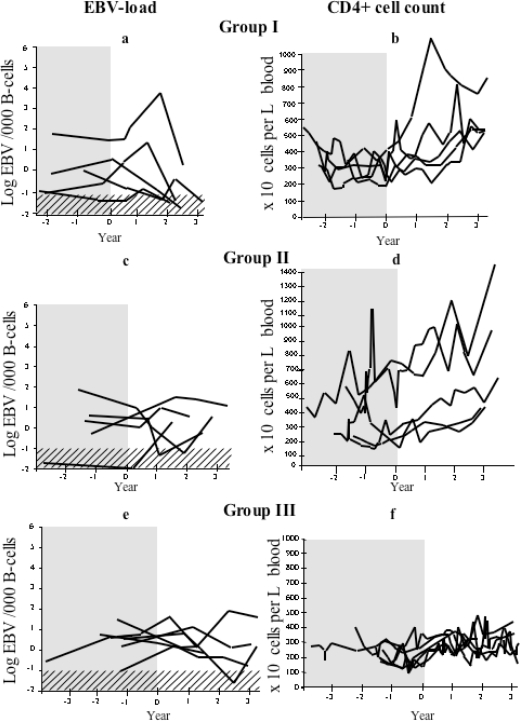
The effect of cART treatment on CD4 counts and EBV load over time. Upper limit of EBV load in healthy controls is stipled and time before cART is shadowed. **(a)** EBV load in group I (with constant undetectable HIV-values after introduction of cART) and **(b)** CD4 levels in group I. **(c)** EBV load in group II (with most but not all HIV-values below detection limit after introduction of cART) and **(d)** CD4 levels in group II. **(e)** EBV load group III (with most HIV-RNA values above detection limit after introduction of cART) and **(f)** CD4 levels group III.

After 1–2 years of cART treatment, all patients in group I (n = 5) showed a decrease in the EBV-load, which approached levels seen in healthy controls. However, some group I patients showed a temporary increase of EBV-DNA load during the second year after the initiation of cART (Figure 1a).

The patients in group I showed increased median CD4+ cell counts: from 285 × 10^6^/L during the year before cART (subsequently called pre-treatment levels) to 530 × 10^6^/L (p < 0.05) at the end of the study (Figure 1b).

**Figure 2.**
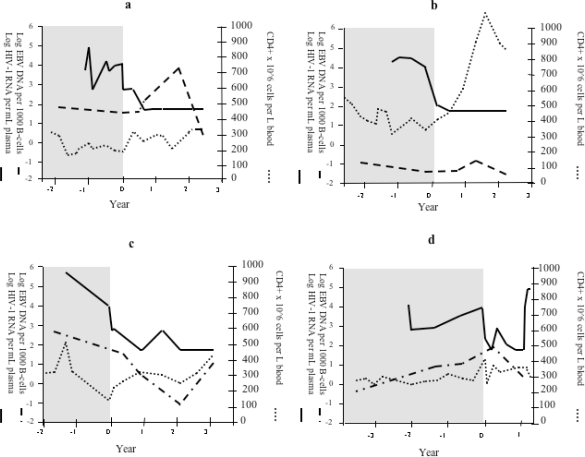
Examples of individual responses of patients from all three groups. EBV load (10-log) (dashed line), HIV-RNA titer (10-log) (solid line), and CD4+ cell count (dotted line) over time. Time 0 is defined as the start of cART. Time before cART is shadowed. Panel **(a)** and **(b)** represent two patients from group I. Patient A had CD4+ cell count of 200 × 10^6^/L the year before cART; patient B had a median CD4+ cell count of 380 × 10^6^/L the year before cART. **(c)** Example of a patient from group II. **(d)** Example of a patient from group III.

Figures 2a and 2b exemplify two individuals from group I. These two patients differed in their initial CD4 status: one had a median pre-treatment CD4+ cell count of 200 × 10^6^/L and the other a median pre-treatment CD4+ count of 380 × 10^6^/L. In the more immunodeficient patient, the EBV control seemed initially absent and an initial increase of the EBV load was seen. However, after two years of cART treatment the levels of the EBV load decreased. The second patient, with higher initial CD4+ cell counts, displayed an EBV load comparable to that seen in healthy individuals both before and during cART treatment.

Patients in group II (n = 5) showed a continuous improvement of CD4 cell counts without restoration of EBV control, *i.e.*, no decrease of the EBV load (exemplified in Figure 2c). In these patients, the CD4+ cell counts increased during treatment from a median pre-treatment value of 180 × 10^6^/L to 570 × 10^6^/L (p < 0.05) at the end of the study. In this group, the two patients with the highest pre-treatment CD4+ cell counts had a slightly lower EBV load at the end of observation time than before treatment, while the others showed a variable pattern with no consistent decrease in the EBV load (Figure 1c respectively Figure 1d).

**Figure 3.**
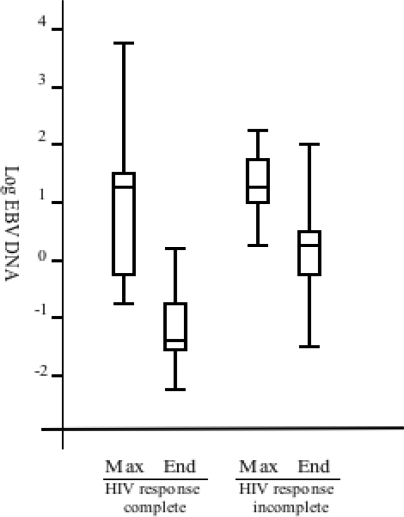
Box plot representing median values and distribution of EBV load in patients grouped according to HIV-RNA response after initiation of cART. Maximum EBV load and EBV load at the end of the study (>1.5 years after introduction of treatment initiation) in relation in groups I and III. The boxes represent 50% of patients in first quartile to third quartile. Bars under and above the boxes represent distribution of all the values. The horizontal line marks the median.

**Figure 4.**
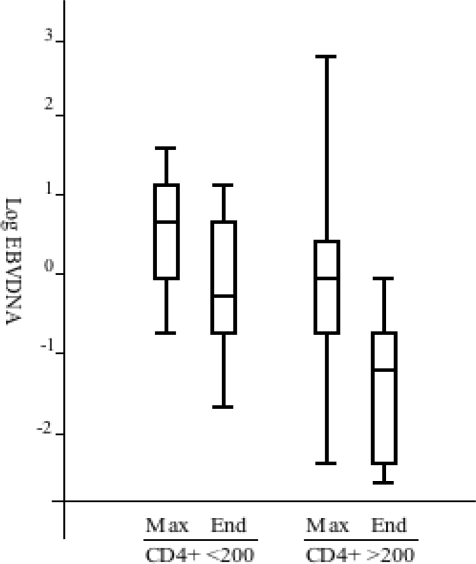
Box plot representing median values and distribution of EBV load in patients grouped according to median CD4 values and patient grouping. A) Maximum EBV load (Max) and EBV load at one year after introduction of cART (End), respectively, in patients with CD4+ cell count median values <200 × 10^6^/L (n = 6) and ≥ 200 × 10^6^/L (n = 13) during the 12 months before introduction of cART. The boxes represent 50% of patients in first quartile to third quartile. Bars under and above the boxes represent distribution of all the values. The horizontal line marks the median.

In group III (n = 6), the EBV load was high and showed a fluctuation, which indicated poor virological and immunological control (Figure 1e). Only a moderate increase in CD4+ cell counts was observed: from the median pre-treatment value of 200 × 10^6^/L to 240 × 10^6^/L at the end of the study (Figure 1f). This might be due to an incomplete response to treatment (as exemplified in Figure 2d). For more than one year preceding the initiation of treatment with protease inhibitor (PI) or other drugs from the non-nucleoside analog reverse transcriptase inhibitors (NRTIs) category, all group III patients had been treated with one or two NRTIs. Four of these patients showed an increase in the EBV-DNA levels during study period. The effect on EBV load is summarized in Figure 3. We further analyzed EBV response in relation to CD4+ levels before treatment (Figure 4). For this purpose, the patients with sufficient number of CD4-count measurements were divided in two groups according to the median pre-treatment CD4+ cell count (below and above 200 × 10^6^/L). There were six and 13 patients in the low and high CD4-count groups, respectively. The median peak EBV value, *i.e.*, the highest value detected, was one 10-log higher in the group with low CD4 levels when compared to those with high CD4 levels. The median EBV load one year after the initiation of cART was within normal range in the group with higher pre-treatment CD4 levels, but remained high in the patient group with lower CD4 values.

Although the association between HIV-1 infection and the development of lymphoma was noted early in the epidemic [12,13], the pathogenic mechanisms have not been clarified. Immune suppression as well as a general immune activation is likely to play a crucial role in the pathogenesis [14]. In addition, EBV is considered to be a strong predisposing, or even a causative, factor. Transplant patients represent another group with a high risk of EBV-positive NHL. In these patients, the constant immune modulation with modern immune suppressants in combination with minor mismatches may explain the high NHL risk [15]. EBV load has been suggested as a predictor for risk of lymphoproliferative disease in this group [16–18].

After the introduction of cART in 1996, the incidence of HIV-related NHL has been considerably reduced [19]. In the current study, we observed differences between various groups of cART treated HIV patients in the dynamics of the balance between EBV infection and host response, in relation to the pre-treatment CD4 levels and the efficiency of cART in controlling HIV-RNA levels. We noticed some patients with an incomplete EBV control, who had an adequate effect of cART treatment on the HIV-1 RNA levels. Our results may indicate that an HIV-induced immune dysregulation persists even after reduction of HIV-1 load to undetectable levels [14]. Similar to other studies, we noticed a lack of EBV control in patients with an initially low CD4+ cell count (<200 × 10^6^/L) and labile virological response to cART [20], sometimes resulting in a biphasic EBV response. This phenomenon is similar to hepatitis B, hepatitis C and Varicella-Zoster virus flares, which were earlier described in the process of immune restoration in HIV-patients following cART treatment [21]. It is likely that an increased lymphoma risk remains during the period with high EBV load and probably exists until the establishment of permanent EBV control as also suggested by others [22,23]. In patients with non-successful HIV-therapy this risk may be more pronounced due to the higher EBV DNA levels. The latter phenomenon is exemplified both by patients with most HIV RNA values as well as those with some HIV values just above the detection limit. It is also in line with the most recent international recommendation to initiate antiretroviral therapy when CD4+ cell counts fall below 350 × 10^6^/L to prevent a decrease to values as low as 200 × 10^6^/L. However, consequences of persistent high EBV-DNA load in each individual HIV-infected patient have not yet been elucidated. If high EBV load precedes lymphoma, the results of our study might indicate a need for EBV-monitoring in patients initiating cART with CD4 values below 200 × 10^6^/L and in non-successfully treated HIV-infected patients. Patients at risk for NHL could be identified at an earlier stage by monitoring EBV load, as is routinely done in post-transplant patients [18,24,25]. However, the nature of EBV infection in HIV-positive subjects is very different from that in post-transplant patients. In these (re-)appearance of EBV-specific CD8(+) T cells leads to an immediate decrease in EBV load [26]. This may be due to that the HIV carriers have a continuous exhaustive deregulation of the normal B cell biology and a reduction in immunity plays a role in developing NHL. In post-transplant patients, a modified immunosuppressive therapy or an antiviral EBV-treatment can prevent the development of malignancies from a preceding polyclonal lymphoproliferative disease [24,25]. However, the lymphoma risk did not increase during a long term follow up in a group of HIV-1 infected patients with temporarily high EBV load in PBMC [27]. None of our patients developed lymphoma during the follow up period of two to six years, although some patients showed extremely high EBV load values. This is interesting since a combination of high EBV load with the reduced function of specific EBV-reactive T-cells has been suggested to affect the risk of NHL [10].

Similar to our results, Righetta *et al.* [20] observed an elevated EBV load as well as a biphasic EBV response after cART initiation in a study of highly immunocompromised HIV-1 patients. They also observed a correlation between an increase of EBV-DNA and anti-EA or anti-VCA. In a study by Stevens *et al.* [28], no difference in the EBV load was detected between patients with and without cART. In this study, the median time of cART treatment was only 13 months. According to our observations, this is an insufficient time for re-establishing EBV control. The absence of restored EBV control is also reflected in serologic analysis where anti-EBNA-IgG was reduced and anti-VCA-IgG increased as shown by others [20].

## 3. Experimental

### 3.1. Patients

Twenty HIV-infected patients, selected from a recombinant gp-160 vaccine trial, were followed with repeated analyses of the EBV load, HIV-RNA titer and CD4+ cell counts, at Karolinska University Hospital, Huddinge (KUHH), between 1995 and 2000. At inclusion, the median CD4+ cell count was 255 × 10^6^/L (range 120–480) and the first analysis of HIV-RNA done in the end of 1995 or the beginning of 1996 showed a median value of 37,000 copies/mL (range <500–1,200,000). In Table 1 we summarize the clinical characteristics of the patients. HIV negative controls were recruited among healthy laboratory staff and were not matched for age, sex, or risk group.

The cART treatment was initiated and given independently of this study according to clinical practice.

Blood samples for EBV-analysis (20 mL) were drawn after informed consent at the time of the regular visits to the open HIV-ward at KUHH, with 6–12 months intervals. We collected two to six samples from each patient. In 1999, complete EBV-serology was analyzed using frozen plasma samples collected during the same year from all the patients.

All 20 patients had IgG-antibodies to VCA and to EBNA as a result of the EBV-carrier status. Sixteen of the patients also had detectable anti-EA-titers. While the anti-EBNA titers were within normal range, the anti-VCA and anti-EA titers were elevated in the majority of the patients (anti-VCA GMT: 960, range 1:160–5120; anti-EA GMT: 80, range: 1:20–640). At the time of sampling for serology, the median CD4+ cell count was 365 × 10^6^/L (170–1010). The CD4+ cell counts, HIV-RNA values, and clinical data were collected from patient files.

None of the patients were diagnosed with lymphoma or AIDS and no deaths occurred during the study period. Five patients were on ART at the start of the study (three on dual drug- and two mono-drug therapy). All patients except one received cART during the study period; 18 received combination therapy, with nucleoside reverse transcriptase inhibitor (NRTI) in combination with protease inhibitor and/or a non NRTI. One patient received only a dual NRTI combination, and one patient continued mono-therapy with azidothymidine.

### 3.2. EBV-DNA Analysis

CD19 positive B lymphocytes were isolated according to Ehlin-Henriksson *et al*. [29]. The enriched B cells were suspended in 100 μL PCR-lysate buffer [30]. The samples were then stored at −20 °C and proteinase-K was inactivated by heating for 15 minutes at 94 °C before PCR. PCR amplification of viral DNA was performed according to Gustafsson *et al.* [31] using a set of nested primers specific for the LMP1-promotor and its upstream control sequence (LRS) region (co-ordinates in B-95-8 prototype strain in parentheses): the outer primer pair was LSY: 5′-CCT TTC TAC GCT TAC ATG CAC ACA C-3′ (169 678 to 169 654), and LAY: 5′-TGG ACA GAG AAG GTC TCT TCT GAA G-3′ (169 239 to 169 263); the inner primer pair was LSI: 5′-CTA CAT CCC AAG AAA CAC GCG TTA-3′ (169 586 to 169 561), and LAI: 5′-AAG CAT GAG AGC AAA GGA ATA GAG-3′ (169 290 to 169 314). The EBV genome number was calculated according to Gustavsson *et al*. [31]. The EBV negative cell line BJAB [14], water and lysis buffer without DNA were included as negative controls. Namalwa cells with one EBV genome copy per cell were used as a positive control. [15]. Sensitivity of the PCR-assay was established to one genome.

The method has been validated against Real-Time (RT-) PCR and the results correlated strongly. However RT-PCR occasionally provided no values on clinical samples due to unsatisfactory DNA quality, while this problem was not noticed using our semi-quantitative method.

### 3.3. EBV Serology

Serology for Epstein-Barr virus was tested according to Svahn *et al.* [32]. Titration of serum antibodies to EA and VCA was performed using the EBV-positive cell line B 95-8 as target. Antibodies against EBNA1 proteins were performed using an ELISA against peptide p107 from the EBNA 1 sequence.

### 3.4. Analysis of Lymphocyte Subsets and HIV-RNA

Data on subsets of T-lymphocytes, CD4 and CD8, as well as HIV-RNA were obtained by routine assays performed in a standardized clinical laboratory.

### 3.5. Statistics

The EBV genome numbers were calculated based on the fraction of positive reactions at each dilution according to Reed-Muench [33] and by the Poisson distribution formula, using a method originally designed to determine the precursor frequency of antigen-specific T cells [34].

## 4. Conclusions

In this long term follow up of moderately immune suppressed HIV-infected patients, we observed a high individual variation of EBV-DNA levels. cART induced virological control of the HIV-infection with undetectable HIV-RNA levels seems to be an insufficient prerequisite for an optimal EBV control, as indicated by Gerard *et al.* [35]. CD4+ cell counts may better reflect the restoration of the patient’s control of persisting EBV infection, as suggested by Kostense *et al.* [36]. This reconstitution of the immune system in HIV-infection takes place during one to several years after the introduction of cART, as reflected by the continuous increase in CD4+ cell counts [37]. EBV load may therefore be considered as a qualitative marker of the actual immune competence in cART treated patients, as also suggested for post-transplant patients [18,38]. Although immunological recovery depends on suppression of HIV-replication, the recovery of the EBV-host balance is certainly not an immediate consequence of the achieved control of HIV.

## Figures and Tables

**Table 1.** Demographic patient data and route of transmission ^(1)^.

**Patients**	n
Number of patients	20
Females	3
Median age	40(31–65)
**Route of infection**	
Heterosexual	9
MSM*	10
Unknown	1
**Origin**	
Northern Europe	15
Africa	5

* Men who have sex with men.

(1) Patients were divided in three groups based on HIV-load. There were no differences in gender, median age and route of transmission between the groups. Group I: constant undetectable HIV-values after introduction of cART. Group II: most but not all HIV-values below detection limit after introduction of cART: Group III: almost all HIV-RNA values above detection limit.

## References

[1] Ernberg I (1986). The role of Epstein-Barr virus in lymphomas of homosexual males. Prog Allergy.

[2] Gaidano G, Dalla-Favera R (1995). Molecular pathogenesis of AIDS-related lymphomas. Adv Cancer Res.

[3] Gerritsen EJ, Stam ED, Hermans J, van den Berg H, Haraldsson A, van Tol MJ, van den Bergh RL, Waaijer JL, Kroes AC, Kluin PM, Vossen JM (1996). Risk factors for developing EBV-related B cell lymphoproliferative disorders (BLPD) after non-HLA-identical BMT in children. Bone Mar Transpl.

[4] Lucas KG, Small TN, Heller G, Dupont B, O'Reilly RJ (1996). The development of cellular immunity to Epstein-Barr virus after allogeneic bone marrow transplantation. Blood.

[5] MacMahon EME, Glass JD, Hayward SB, Mann RB, Becker PS, Charache P, McArthur JC, Armbinder RF (1991). Epstein-Barr virus in AIDS-related primary central nervous system lymphoma. Lancet.

[6] Camilleri-Broët S, Davi F, Feuillard J, Bourgeois C, Seilhean D, Hauw JJ, Raphaël M (1995). High expression of latent membrane protein 1 of Epstein-Barr virus and BCL-2 oncoprotein in acquired immunodeficiency syndrome-related primary brain lymphomas. Blood.

[7] Hamilton-Dutoit SJ, Pallesen G, Franzmann MB, Karkov J, Black F, Skinhoj P, Pedersen C (1991). AIDS-related lymphoma. Histopathology, immunophenotype, and association with Epstein-Barr virus as demonstrated by in situ nucleic acid hybridization. Am J Pathol.

[8] Matsuura A (1989). The latent Epstein.Barr virus (EBV) activation and EBV-specific immunological abnormality in human immunodeficiency virus (HIV) infected hemophiliacs. Hokkaido Igaku Zasshi.

[9] Palmieri C, Treibel T, Large O, Bower M (2006). AIDS-related non-Hodgkin's lymphoma in the first decade of highly active antiretroviral therapy. QJM.

[10] van Baarle D, Kostense S, Hovenkamp E, Ogg G, Nanlohy N, Callan MF, Dukers NH, McMichael AJ, van Oers MH, Miedema F (2002). Lack of Epstein-Barr virus- and HIV-specific CD27- CD8+ T cells is associated with progression to viral disease in HIV-infection. AIDS.

[11] Sandström E, Wahren B (1999). Therapeutic immunisation with recombinant gp160 in HIV-1 infection: a randomised double-blind placebo-controlled trial. Nordic VAC-04 Study Group. Lancet.

[12] Herndier BG, Kaplan LD, McGrath MS (1994). Pathogenesis of AIDS lymphomas. AIDS.

[13] Lyter D, Besley D, Thackeray R (1994). Incidence of malignancies in the Multicenter AIDS cohort study (MACS). Proc ASCO.

[14] Leng Q, Borkow G, Weisman Z, Stein M, Kalinkovich A, Bentwich Z (2001). Immune activation correlates better than HIV plasma viral load with CD4 T-cell decline during HIV infection. AIDS.

[15] Grulich AE, Wan X, Law MG, Milliken ST, Lewis CR, Garsia RJ, Gold J, Finlayson RJ, Cooper DA, Kaldor JM (2000). B-cell stimulation and prolonged immune deficiency are risk factors for non-Hodgkin's lymphoma in people with AIDS. AIDS.

[16] Rooney CM, Loftin SK, Holladay MS, Brenner MK, Krance RA, Heslop HE (1995). Early identification of Epstein-Barr virus-associated post-transplantation lymphoproliferative disease. Br J Haematol.

[17] Lucas KG, Burton RL, Zimmerman SE, Wang J, Cornetta KG, Robertson KA, Lee CH, Emanuel DJ (1998). Semiquantitative Epstein-Barr virus (EBV) polymerase chain reaction for the determination of patients at risk for EBV-induced lymphoproliferative disease after stem cell transplantation. Blood.

[18] Bakker N, Verschuuren E, Erasmus M, Hepkema B, Veeger N, Kallenberg C, van der Bij W (2007). Epstein-Barr virus-DNA load monitoring late after lung transplantation: a surrogate marker of the degree of immunosuppression and a safe guide to reduce immunosuppression. Transplantation.

[19] Bonnet F, Chêne G (2008). Evolving epidemiology of malignancies in HIV. Curr Opin Oncol.

[20] Righetta E, Ballon G, Ometto L, Cattelan AM, Menin C, Zanchetta M, Chieco-Bianchi L, De Rosa A (2002). Dynamics of Epstein-Barr virus in HIV-1 infected subjects on highly active antiretroviral therapy. AIDS.

[21] Cooney EL (2002). Clinical indicators of immune restoration following highly active antiretroviral therapy. Clin Inf Dis.

[22] Bower M, Fisher M, Hill T, Reeves I, Walsh J, Orkin C, Phillips AN, Bansi L, Gilson R, Easterbrook P, Johnson M, Gazzard B, Leen C, Pillay D, Schwenk A, Anderson J, Porter K, Gompels M, Sabin CA, for the UK CHIC Steering Committee (2009). CD4 counts and the risk of systemic non-Hodgkin’s lymphoma in individuals with HIV in the UK. Haematologica.

[23] Engels EA, Pfeiffer RM, Landgren O, Moore RD Immunologic and Virologic Predictors of AIDS-Related Non-Hodgkin Lymphoma in the Highly Active Antiretroviral Therapy Era. J Acquir Immune Defic Syndr.

[24] Starzl T, Nalesnik M, Porter K, Ho M, Iwatsuki S, Griffith B, Rosenthal J, Hakala T, Shaw B, Hardesty R (1984). Reversibility of lymphomas and lymphoproliferative lesions developing under cyclosporin-steroid therapy. Lancet.

[25] Pirsch JD, Stratta RJ, Sollinger HW, Hafez GR, D'Alessandro AM, Kalayoglu M, Belzer FO (1989). Treatment of severe Epstien-Barr virus-induced lymphoproliferative syndrome with Ganciclovir: Two cases after solid organ transplantation. Am J Med.

[26] Pietersma F, Piriou E, van Baarle D (2008). Immune surveillance of EBV-infected B cells and the development of non-Hodgkin lymphomas in immunocompromised patients. Leuk Lymphoma.

[27] van Baarle D, Wolthers KC, Hovenkamp E, Niesters HGM, Osterhaus ADME, Miedema F, van Oers MHJ (2002). Absolute Level of Epstein-Barr Virus DNA in Human Immunodeficiency Virus Type 1 Infection Is Not Predictive of AIDS-Related Non-Hodgkin Lymphoma. J Inf Dis.

[28] Stevens SJC, Blank BSN, Smits PHM, Meenhorst PL, Middeldorp JM (2002). High Epstein-Barr virus (EBV) DNA load in HIV-infected patient: correlation with antiretroviral therapy and quantitative EBV serology. AIDS.

[29] Ehlin-Henriksson B, Zou JZ, Klein G, Ernberg I (1999). Epstein-Barr virus genomes are found predominantly in IgA-positive B cells in the blood of healthy carriers. Int J Cancer.

[30] Albert J, Fenyö EM (1990). Simple, sensitive and specific detection of HIV-1 in clinical specimens by polymerase chain reaction with nested primers. J Clin Microbiol.

[31] Gustafsson A, Levitsky V, Zou JZ, Frisan T, Dalianis T, Ljungman P, Ringden O, Winiarski J, Ernberg I, Masucci MG (2000). Epstein-Barr virus (EBV) load in bone marrow transplant recipients at risk to develop posttransplant lymphoproliferative disease: prophylactic infusion of EBV-specific cytotoxic T cells. Blood.

[32] Svahn A, Magnusson M, Jagdahl L, Schloss L, Kahlmeter G, Linde A (1997). Evaluation of three commercial enzyme-linked immunosorbent assays and two for diagnosis of primary Epstein-Barr virus infection. J Clin Microbiol.

[33] Reed LJ, Muench H (1938). A simple method of estimating fifty per cent endpoints. An J Hyg.

[34] Waldman H, Cobbold S, Lefkovits I, Klaus GGB (1987). Limitid dilution analysis. Lymphocytes: a Practical Approach.

[35] Gérard L, Meignin V, Galicier L, Fieschi C, Leturque N, Piketty C, Fonquernie L, Agbalika F, Oksenhendler E (2009). Characteristics of non-Hodgkin lymphoma arising in HIV-infected patients with suppressed HIV replication. AIDS.

[36] Kostense S, Otto SA, Knol GJ, Manting EH, Nanloh NM, Jansen C, Lange JM, van Oers MH, Miedema F, van Baarle D (2002). Functional restoration of human immunodeficiency virus and Epstein-Barr virus-specific CD8+ T cells during highly active antiretroviral therapy is associated with an increase in CD4+ T cells. Eur J Immunol.

[37] Kaufmann GR, Bloch M, Finlayson R, Zaunders J, Smith D, Cooper DA (2002). The extent of HIV-1 related immunodeficiency and age predict the long-term CD4 T lymphocyte response to potent antiretroviral therapy. J Acquir Immune Defic Syndr.

[38] van Esser JWJ, Niesters HGM, van der Holt B, Meijer E, Osterhaus ADME, Gratama JW, Verdonck LF, Löwenberg B, Cornelissen JJ (2002). Prevention of Epstein-Barr virus–lymphoproliferative disease by molecular monitoring and preemptive rituximab in high-risk patients after allogeneic stem cell transplantation. Blood.

